# Improving Resident Doctors’ Preparedness in Psychiatric Intensive Care: A Teaching-Based Quality Improvement Initiative

**DOI:** 10.1192/bjo.2026.11486

**Published:** 2026-06-30

**Authors:** Aashna Singh, Satyam Kishore

**Affiliations:** Tees, Esk and Wear Valleys NHS Foundation Trust, Middlesbrough, United Kingdom

## Abstract

**Aims:**

This quality improvement project aimed to improve resident doctors’ self-reported confidence and preparedness for managing clinical, legal, and safety-related aspects of Psychiatric Intensive Care work through a structured teaching intervention.

**Methods:**

A scenario-based teaching session titled *Safe Working on PICU* was developed and delivered across two teaching cycles (February and September 2025). Anonymised pre- andpost-session questionnaires assessed confidence across key domains using a 1–5 Likert scale and free-text feedback. Iterative refinement of the teaching content was guided by Plan–Do–Study–Act (PDSA) methodology.

**Results:**

Across two teaching cycles, improvements in self-reported confidence were observed in 6 of 7 assessed domains. Mean confidence scores increased by between 0.2 and 0.65 points on a 5-point Likert scale, with the largest gains seen in understanding of seclusion processes, Intensive Care Unit roles, management of aggression, and multidisciplinary collaboration. Confidence relating to Section 5(2) showed a small reduction following more detailed legal teaching, interpreted as increased awareness of legal complexity. Qualitative feedback consistently highlighted improved clarity, relevance to on-call practice, and the value of case-based discussion.

**Conclusion:**

A structured, scenario-based teaching intervention can meaningfully improve resident doctors’ perceived preparedness for Psychiatric Intensive Care work. Teaching-based QI offers a practical approach to addressing safety-critical learning needs in high-acuity psychiatric settings, with potential for wider implementation and sustainability.

